# Prenatal Torsion Of Testis: A Rare Emergency

**Published:** 2012-04-01

**Authors:** Neha Singh Shrivastava, Chandrasekhar Gopalaswamy, Ram Mohan Chivukula Venugopal

**Affiliations:** Department of Pediatrics, Cosmopolitan Hospital, Thiruvananthapuram, Kerala, India; 1 Department of Pediatric Surgery, Cosmopolitan Hospital, Thiruvananthapuram, Kerala, India

**Dear Sir,**

Prenatal torsion of testis is an extremely rare surgical emergency with controversial management guidelines. Controversy exists not only with regard to timing and necessity of exploration but also whether or not to fix the contralateral testis as there is no predisposing anatomical defect. Though not life-threatening, it risks fertility of an otherwise healthy newborn male if overlooked. We describe a case of prenatal testicular torsion and discuss pertinent issues.

A term male newborn was found to have edematous and enlarged right hemiscrotum at birth. General condition of neonate was satisfactory with a birth weight of 2.75 kg. Vital signs were within normal range. Systemic examination revealed no abnormality except for the enlarged, edematous and erythematous right hemiscrotum (Fig. 1). On palpation, right testis was enlarged, mildly tender, firm in consistency and at a higher position than the left, but with no local rise of temperature. Scrotal transillumination was reduced on the right side. Left testis was in descended position with appropriate size and consistency. Clinical diagnosis of testicular torsion was made. Immediate color doppler ultrasound study of scrotum showed enlarged, heterogeneous right testes, with focal hypoechoic region (suggestive of infarct), no visible internal vascularity, minimal peritesticular fluid and scrotal wall edema; indicating antenatal torsion of the testis. Left testis was found to have normal size, echogenicity and internal vascularity. Since these findings were suggestive of an irreversible ischemia and prolonged duration of torsion, emergent exploration was deferred and decision was taken to manage the case electively.

**Figure:**
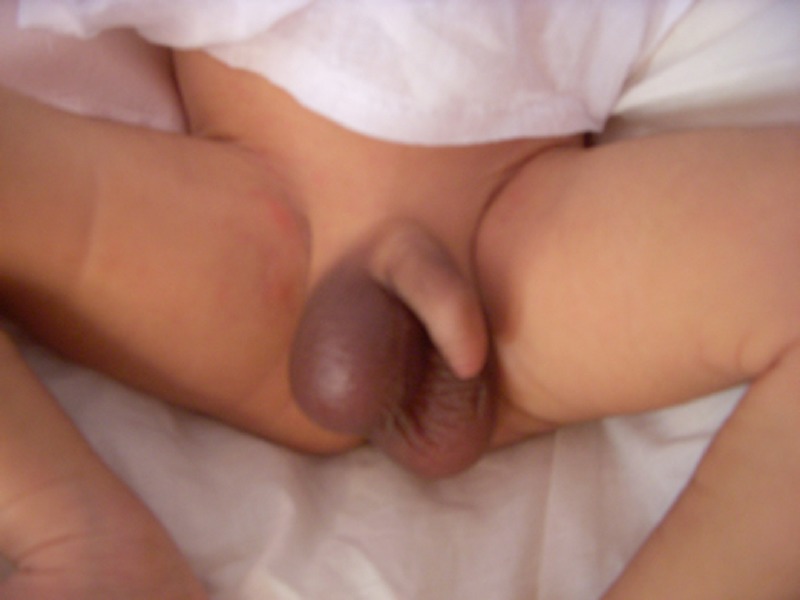
Figure 1: Newborn with prenatal torsion of testis (right sided).

After detailed counseling of parents regarding the objectives and risks of exploration, including the low likelihood of salvaging the affected testis, patient was promptly explored after adequate pre-anaesthetic workup. Surgery under general anaesthesia on 3rd post natal day revealed extravaginal type spermatic cord torsion through two complete turns with gangrenous testes on the affected side. Hence right orchidectomy and left orchidopexy were done using separate scrotal incisions. Histological study of excised testis showed hemorrhagic and gangrenous changes with no viable testicular tissue. Post operative period was uneventful and the neonate is doing well on follow-ups.

Prenatal testicular torsion typically presents as a discoloured scrotum seen at birth in an otherwise asymptomatic newborn with decreased scrotal transillumination. Other presentations may be as testicular nubbin, small and hard testis, normal-sized but hard testis or as acute scrotum. Bilateral torsion can occur in up to 20% of affected newborns and is often an asynchronous event [1]. Worryingly, bilateral torsion can present with unilateral signs [2].

Doppler ultrasound is an important tool for diagnosing testicular torsion and predicting the viability of testis. Presence of any blood flow suggests incomplete torsion and the potential for salvage. Highest recovery rates have been found in testes with normal, homogeneous echogenicity as compared to no recovery in testes with heterogeneous echogenicity [3].

Management of unilateral prenatal torsion is an area of controversy with strategies including observation alone, delayed contralateral orchidopexy and emergent contralateral orchidopexy [1]. Elective orchidectomy with contralateral orchidopexy once anaesthesia risk assessment has been stabilized (within 1-2 days of diagnosis), has been the commonly used and preferred approach [4]. But as bilateral testicular torsion is now being reported with more frequency and in view of recent experiences with lack of significant contralateral symptoms in cases of bilateral asynchronous torsions, many authors now recommend emergent surgical exploration in all cases of prenatal torsions [1,2]. Studies have shown testicular salvage rate of 5% in cases of prenatal torsion [1]. With this low likelihood of salvage, theoretical risk of contralateral torsion and anorchia must be weighed against the low but real risk of general anesthesia in a healthy newborn.

Bilateral torsion is a true emergency and the management is straightforward immediate surgery. Threshold for performing bilateral testicular detorsion and fixation should be low and every effort should be made to leave even necrotic testes in place, as some testicular function may still be possible [5].

Prompt recognition and rapid intervention is the key to preserve testicular function in cases of torsion. As salvage rates are poor for prenatally torted testis, prevention of contralateral testicular torsion is imperative. Hence early exploration with contralateral orchidopexy is highly recommended. Doppler ultrasound is useful however any suspicion of bilateral involvement and incomplete torsion should warrant immediate exploration.

## Footnotes

**Source of Support:** Nil

**Conflict of Interest:** None declared

